# A genome-wide identification and analysis of the DYW-deaminase genes in the pentatricopeptide repeat gene family in cotton (*Gossypium* spp.)

**DOI:** 10.1371/journal.pone.0174201

**Published:** 2017-03-24

**Authors:** Bingbing Zhang, Guoyuan Liu, Xue Li, Liping Guo, Xuexian Zhang, Tingxiang Qi, Hailin Wang, Huini Tang, Xiuqin Qiao, Jinfa Zhang, Chaozhu Xing, Jianyong Wu

**Affiliations:** 1 State Key Laboratory of Cotton Biology (China)/Institute of Cotton Research of Chinese Academy of Agricultural Science, Anyang, Henan, China; 2 Department of Plant and Environmental Sciences, New Mexico State University, Las Cruces, New Mexico, United States of America; USDA-ARS Southern Regional Research Center, UNITED STATES

## Abstract

The RNA editing occurring in plant organellar genomes mainly involves the change of cytidine to uridine. This process involves a deamination reaction, with cytidine deaminase as the catalyst. Pentatricopeptide repeat (PPR) proteins with a C-terminal DYW domain are reportedly associated with cytidine deamination, similar to members of the deaminase superfamily. PPR genes are involved in many cellular functions and biological processes including fertility restoration to cytoplasmic male sterility (CMS) in plants. In this study, we identified 227 and 211 DYW deaminase-coding PPR genes for the cultivated tetraploid cotton species *G*. *hirsutum* and *G*. *barbadense* (2n = 4x = 52), respectively, as well as 126 and 97 DYW deaminase-coding PPR genes in the ancestral diploid species *G*. *raimondii* and *G*. *arboreum* (2n = 26), respectively. The 227 *G*. *hirsutum* PPR genes were predicted to encode 52–2016 amino acids, 203 of which were mapped onto 26 chromosomes. Most DYW deaminase genes lacked introns, and their proteins were predicted to target the mitochondria or chloroplasts. Additionally, the DYW domain differed from the complete DYW deaminase domain, which contained part of the E domain and the entire E+ domain. The types and number of DYW tripeptides may have been influenced by evolutionary processes, with some tripeptides being lost. Furthermore, a gene ontology analysis revealed that DYW deaminase functions were mainly related to binding as well as hydrolase and transferase activities. The *G*. *hirsutum* DYW deaminase expression profiles varied among different cotton tissues and developmental stages, and no differentially expressed DYW deaminase-coding PPRs were directly associated with the male sterility and restoration in the CMS-D2 system. Our current study provides an important piece of information regarding the structural and evolutionary characteristics of *Gossypium* DYW-containing PPR genes coding for deaminases and will be useful for characterizing the DYW deaminase gene family in cotton biology and breeding.

## Introduction

RNA editing is a post-transcriptional process affecting organellar transcripts that results in nucleotide changes from DNA to RNA. There are various types of RNA editing in different organisms. In mammals, a zinc-dependent enzyme related to cytidine deaminase is responsible for converting cytidines (C) to uredines (U) in *apoB* mRNA [1, 2]. In vascular plants, RNA editing occurs in chloroplasts and mitochondria, and almost exclusively involves C-to-U changes [2]. Additionally, nuclear genes encoding proteins involved in RNA editing belong to the gene subfamily associated with pentatricopeptide repeat (PPR) proteins.

The PPR protein family is one of the largest protein families in higher plants [3]. The PPR proteins usually have 2–27 repeating motifs consisting of 35 amino acids arranged in clusters [3, 4]. These motifs are divided into three classes. The canonical PPR motifs (i.e., P motifs) are common among eukaryotes, while the short PPR-like (i.e., S motifs) and long PPR-like motifs (i.e., L motifs) are present only in higher plants [4]. The PPR protein family is divided into two subfamilies (i.e., P and PLS subfamilies) based on the different motifs. The P subfamily members contain only the P motif, while the PLS subfamily members usually consist of a tandem array of degenerated P, L, and S motifs. The PLS subfamily proteins are plant-specific and usually contain an additional C-terminal extension with E, E+, and DYW domains. According to the C-terminal extension, this subfamily can be divided into the following four subgroups: PLS, E, E+, and DYW [4]. *Arabidopsis thaliana* carries 87 genes encoding PPR proteins with the DYW domain. The DYW domain is named for the frequent presence of an Asp–Tyr–Trp tripeptide at the C-terminal, and has been detected only in the proteins of upland plants. The E and E+ domains are usually highly degraded and difficult to identify. The DYW domain contains a highly conserved region with α-β-α secondary structure consisting of conserved amino acids (e.g., Cys and His) [5].

The DYW domain is the candidate catalytic domain for cytidine deaminase. The HxExx…CxxCH motifs in the DYW domain align with the active site of cytidine deaminase (i.e., C/HxExx…xPCxxC). Additionally, the DYW domain is highly evolutionarily correlated with RNA editing. Thus, proteins containing the DYW domain have been classified as deaminases involved in RNA editing [6, 7]. As such, proteins are grouped into the nucleic acid deaminase superfamily (i.e., DYW deaminase family; Pfam: Pf14432) according to DYW domain characteristics. The DYW deaminase domain contains part of the E domain, the whole E+ domain, and most of the DYW domain [7]. Recently, Hayes et al. (2015) suggested that the conserved Glu residue of the HXE motif in the DYW domain is necessary for RNA editing. When the codon for the Glu residue in the DYW domain of OTP84 and CREF7 was mutagenized, the transgenic plants containing the mutated genes could not efficiently edit the cognate editing sites [8]. Wagoner et al. (2015) proposed that the DYW domain and the E (or E+) domain are essential for cytidine deaminase activity because truncating either domain resulted in a QED1 protein that completely lacked any editing activity. In contrast, the DYW domain was revealed to be unnecessary for four RNA editing factors (i.e., CRR22, CRR28, OTP82, and ELI1) [9]. The DYW deaminase domain is different from the DYW domain in PPR proteins, which is essential for the RNA editing activity of some enzymes but not for others. Thus, the role of the DYW domain during RNA editing has not been fully characterized. Boussardon et al. (2012) suggested that the interaction between CRR4 and DYW1 contributes to the editing of the ndhD-1 site. The DYW domain, which functioned as the catalytic domain, may have been lost in CRR4, which now requires the DYW domain from DYW1 to provide catalytic activity. The DYW1 protein carrying the E+ and DYW domains may interact with CRR4 to edit the ndhD C2 site [10]. In this case, CRR4 may use the DYW1 as the catalytic domain. In summary, as one of the most specific subgroups of the PPR protein family, the DYW deaminase domain-containing PPR proteins are particularly striking because of their RNA editing activities.

Cotton is an economically important fiber crop as the source of the most important natural textile fiber. The genus *Gossypium* contains approximately 45 diploid species and five polyploid species. Crossing between the extant of diploid species *G*. *arboreum* and *G*. *raimondii* and chromosome doubling 1–2 million years ago resulted in the emergence of allotetraploid cotton species, including cultivated *G*. *hirsutum* (accounting for ca. 95% world cotton production) and *G*. *barbadense* [11]. The genomes of the two ancestral diploids (i.e., *G*. *arboreum* and *G*. *raimondii*) and the two tetraploids (i.e., *G*. *hirsutum* and *G*. *barbadense*) have recently been sequenced, providing an opportunity to study cotton gene families. PPR genes including these responsible for fertility restoration to cytoplasmic male sterility (CMS) have been cloned with functions analyzed in several plant species. For instance, *Rf4* encoded a PPR protein for wild abortive-type CMS of rice, was confirmed to reduce the *orf352*-containing transcripts to restore pollen fertility [12], and a PPR protein Rf6 function with hexokinase 6 (OsHXK6) to promote the processing of the aberrant CMS-associated transcript *atp6-orfH79* to rescue HL-CMS of rice [13]. In sorghum, two PPR genes were identified for fertility restoration of A1 cytoplasm [14, 15]. However, no such information is currently available in cotton. In this study, we performed an *in silico* analysis of the four *Gossypium* genome sequences to identify PPR genes encoding for the DYW-containing deaminases. We focused on gene structure and expression with a specific focus on CMS and fertility restoration in *G*. *hirsutum*. The results of this study will contribute to the understanding of the distribution and functions of DYW deaminase genes in cotton.

## Materials and methods

### Materials

The *Gossypium hirsutum* variety Zhong10 was used for different tissue expression analysis. Materials were planted under greenhouse. Roots, stems and leaves were collected in one-month-old seedlings, flowers were collected from plants grown two-month-old on the day of flowering, all tissue samples were obtained from 3 individual plants. Cytoplasm male sterility (CMS-D2) A S(rf1rf1) with cytoplasm from *Gossypium harknessii* and its isogenic restorer line R S(Rf1rf1) were planted under normal conditions in the farm. Floral buds about 3 mm in length (nearly represent the stage of male meiosis) were collected from the near isogenic line with three biological replicates as previous studies, respectively [16, 17]. All collected samples were immediately frozen in liquid nitrogen and stored at -76°C before use. Above the materials were provided by Cotton Research Institute (CRI), Chinese Academy of Agricultural Science (CAAS), Anyang, Henan, China.

### Local databases of sequence data and RNA-seq

Local databases including genome sequence and annotation information files of *Gossypium* species (*G*. *raimondii*, *G*. *arboreum*, *G*. *hirsutum* and *G*. *barbadense* (xinhai)) and other organisms (*Cyanidioschyzon merolae*, *Physcomitrella patens*, *Selaginella moellendorffii*, *Sorghum bicolor*, *Oryza sativa*; Japonica Group) were established. The genome sequence and annotation information of *Gossypium* species was downloaded from the Cottongen (https://www.cottongen.org/). Other organism genome information was downloaded from the NCBI. Arabidopsis thaliana genome sequencewere downloaded from TAIR (http://www.arabidopsis.org/). The extraction of total RNA from flower buds about 3 mm in length (at roughly the stage of male meiosis) of CMS cotton (CMS-D2) A and its isogenic restorer line R was performed by using Spectrum™ Plant Total RNA Kit according to the manufacturer’s guide. RNA concentration was measured using NANODROP 2000. Six transcriptome libraries (A1-3, R1-3) were constructed by using equal amounts of RNA from the three biological replicates. The libraries preparation and sequencing were performed following standard Illumina methods on the Illumina sequencing platform HiSeq™ 2500 system (2×150bp read length). All of the raw sequence data from the Illumina sequencing platform can be found in the Short Read Archive (SRA) database of the National Center for Biotechnology Information (NCBI) under accession number SRX2578795. The FPKMs (mean value of three biological replicates) of gene was further used to perform a differential expression analysis. The criterion was set as: fold change = |log_2_ (sample1/sample2) | ≥ 1 and P ≤ 0.05. The differentially expressed genes between CMS cotton (CMS-D2) A and its isogenic restorer line R were shown in S1 Table. RNA-seq data of gene expression during fiber development stages at 5, 10, 20, 25 dpa and in root, stem, leaf, petal, stamen, and pistil in *G*. *hirsutum* were downloaded from ccNET database (http://structuralbiology.cau.edu.cn/gossypium/) [18].

### DYW-deaminase proteins sequence identification and domain analysis

Download hidden Markov model information (PF14432) of the unique DYW deaminase domain from Pfam29.0 database, then use Pfam file PF14432 to retrieve annotated protein sequences file of *Gossypium* species and other organisms with Hmmsearch program (E value as the default) in the HMMER 3.0 software, obtained sequences above the threshold from totally protein sequences. Domain analysis manually one by one on candidate protein sequences were performed by using online software SMART (http://smart.embl-heidelberg.de/), removed these sequences without DYW deaminase domain.

### Genome wide synteny analysis of DYW deaminase genes

A comparative syntenic map of the DYW deaminase genes from tetraploid cotton species and two diploid cotton species was constructed by using circos-0.69–3 software package, with parameters set as the default.

### Conserved domain alignment and analysis of DYW deaminase proteins in *Gossypium*

By using MEME online software analysis, DYW deaminase protein sequences was used to obtain consensus sequence and Logo of DYW domain and DYW deaminase domain, and compared consensus sequence with tomato and *Arabidopsis thaliana*.

### Phylogenetic tree construction, gene structure analysis, chromosomal and subcellular location of DYW-deaminase proteins

The multiple Sequence alignment of DYW deaminase domain sequence in four cotton species was accomplished by using ClustalX2 software with parameters set as default; the nonroot phylogenetic tree was constructed by the neighbour joining tree (NJ), the bootstrap set for 1000 replications in MEGA 6 software.

According to GFF information, the gene structure display system of Peking University (http://gsds.cbi.pku.edu.cn/) was used to draw the gene structure and DYW deaminase gene were mapped on chromosomes by using Mapchart software.

Using the signal peptide prediction program Target P to predict the subcellular location of DYW-deaminase proteins.

### The GO and expression analysis of DYW-deaminase genes

GO analysis of DYW-deaminase genes completed by blast2go software, and the picture was drawn by online software WEGO (http://wego.genomics.org.cn/cgi-bin/wego/index.pl).

The extraction of RNA from root, stem, leaf and flower was performed using RNAprep Pure Plant kit (TIANGEN, China). Equal amounts of RNA from three biological replicates were equally pooled together and constructed the total RNA of each sample. Reverse transcription was conducted by using 0.5 μg total RNA with PrimeScript^TM^RT reagent kit (Takara Bio Inc., Dalian, China) to 10 μl reaction volume following the manufacturer’s guides and diluted to 100 μl before use. For real-time PCR, 1 μl diluted cDNA was mixed with 10 μl 2 × SYBR Green Mix (Takara) and 1.2 μl primers in a total volume of 20 μl. The PCR was performed using SYBR Green as fluorescence dye and run on Eppendorf Real plex system with melt curve program. The PCR amplification reaction was performed as follows: 94°C for 3 min, 40 cycles of 94°C for 5 s, 56°C for 20 s and 72°C for 25 s, then ending with the melting curve. *Ghhis3* was used as the reference gene for normalization. All reactions were done in three biological replicates, the melting curve was used for each qRT-PCR to determine PCR performance, Ct value in three biological replicates of each gene in four tissues was calculated by 2-△△Ct method [19]. Primers of 16 selected DYW deaminase genes were designed through Primer Premier 5.0 (Table 1).

**Table 1:** The primers of 16 selected PPR DYW deaminase genes for quantitative RT-PCR.

Gene ID	Forward primer(5'- 3')	Reverse primer(5'- 3')
*Gh_D04G0152*	CTCCCCTTTGGATTCTCGTT	ACCAAGTAAACCCAACTTCGC
*Gh_D09G0761*	AAATGCCTGGACGGAATGTT	AACCGCCTCATCAACTCTACC
*Gh_A02G0419*	TCCCATCACCTAACATCGTCTC	TGGTTGGGGAGAATAGGGTC
*Gh_D08G2381*	TTTACATGGTCGAGGAAGGGA	TGAAGTGCTCAACTCCAGGCT
*Gh_D05G0911*	AAGCAGGTCTACTGGAAAACGC	CGTATATTTTTTCAGATTCGGGGTG
*Gh_A03G1665*	TGTCGGAACCTGCCTCATT	TGACCCGCTTCAACTAAACCT
*Gh_A01G1752*	CTTGTTCAAAATTGGGTGCC	TGCTTTCTTGCCTTGACCAT
*Gh_A07G1662*	TGTTATCAGTGCTTGTGCGGG	CCATTCTTACTGCCCCTGCT
*Gh_A12G0594*	AACAAACCCGAAACAGCCC	CATCAAGTTCCAAGAAACGACG
*Gh_D05G0556*	TTGCTCTTATGAATACTCCACCAGG	CCTTGAAATGGTGAAACCGACT
*Gh_D02G1741*	GTTACTTGAAGACGGGCGACA	GTTTGCTTGATTACTCCCCGTT
*Gh_D01G0153*	TGGACCCTGATGATACTGCG	TCTTGCACCCATCACGCTTC
*Gh_A02G0212*	TGGTCGTTGATTTGTTAGGCAG	GCTCCCAGGATGTTTAGGTTCA
*Gh_A12G0486*	GCATACGGATGTAGAGGATTTGG	ACGGCAGACCTGGCTGTTAT
*Gh_A13G1386*	AACCGAATGGAGGGCGTAA	CCGATGTCCCACAAAGTTCA
*Gh_A01G1103*	GGGAAACTCTTCATGCTTACACC	TGCTGCTTTACCTTGACCGT

## Results

### Identification and structural analysis of *Gossypium* genes encoding DYW deaminases

A total of 227 *G*. *hirsutum* genes were identified as encoding DYW deaminases belonging to the PPR family. Most of these proteins contained multiple PPR domains, and 190 proteins contained intact DYW deaminase domain structures. An analysis of the 227 predicted *G*. *hirsutum* DYW deaminases indicated that the number of amino acids in the protein family ranged from 52 to 2016. Furthermore, we identified 126, 97, and 211 DYW deaminases in *G*. *raimondii*, *G*. *arboreum*, and *G*. *barbadense*, respectively. Information about the DYW deaminase genes in *Gossypium* is summarized in S2 Table. All of the DYW deaminases in the four sequenced cotton species had similar protein structures and contained the DYW deaminase domain. Additionally, most of the DYW deaminases also contained PPR domain tandem repeats. Most of the PPR genes had no introns, which is also an important structural feature of the sequences in DYW deaminase genes. In *G*. *hirsutum*, 81% of the genes lacked an intron, while 12% contained only one intron and 7% consisted of two or more introns (S1 and S2 Figs). Similar proportions were observed in *G*. *raimondii* (i.e., 75, 16, and 9%, respectively) and *G*. *arboreum* (i.e., 81, 12 and 7%, respectively). However, in *G*. *barbadense*, only 47% of the DYW deaminase genes lacked introns, while the proportion of genes with introns was relatively high (i.e., 24% with one intron and 27% with two or more introns).

### Conserved sequences in the DYW and DYW deaminase domains

**Fig 1:**
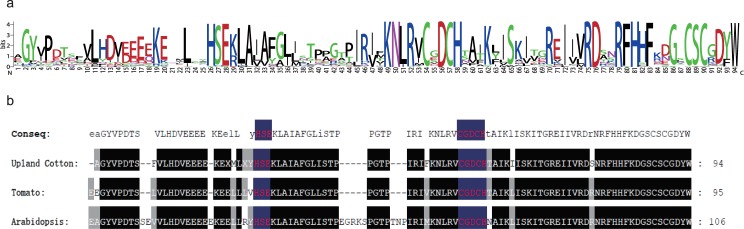
a: The Logo of DYW domain in *G*. *hirsutum*. The higher the letter, the higher the conservation; b: The comparison of DYW domain consensus sequence in upland cotton, tomato and *Arabidopsis*. The capital letters represent highly conserved, lowercase letters indicate lower conservative.

All of the 227 analyzed Upland cotton protein sequences contained the DYW deaminase domain. Additionally, 190 proteins contained a complete and conserved DYW domain, while the remaining 37 proteins contained only a degenerated E+ domain or part of the DYW domain. Using Upland cotton as an example, we obtained the consensus sequences and motif logos for the DYW domains of 68 randomly selected *G*. *hirsutum* protein sequences using the MEME online program. We also compared the sequences of these proteins with the DYW domain consensus sequences from tomato and *Arabidopsis thaliana* (Fig 1). The obtained consensus sequence of the *G*. *hirsutum* DYW domain was as follows: AGYvPDTSFVLHDveEEeKEXMLXYHSEkLAiAFGlisTPpGTPIRifKNLRVCGDCHtAiKlISKItGREIiVRDsNRFHHFKdGSCSCGDYW.

A comparison of the domain sequences revealed that the DYW domains of the above three different species were similar (Fig 1). The sequences contained conserved HxEx…CxxCH motifs, which corresponded to the cytidine deaminase active site (i.e., C/HxEx…CxxC). Cytidine deaminase is a Zn-dependent enzyme in which the CxxCH motif functions as a binding site [20]. The conserved His and Cys residues in the DYW domain combine with Zn ions, while the conserved Glu is required for deamination [8]. Thus, the DYW domain may be involved in nucleotide deaminations. Additionally, a previous study revealed that if CxxCH in the DYW domain mutated into GxxGH, the RNA cleavage activity decreases significantly [5], suggesting that the CxxCH motif in the DYW domain is necessary for endonuclease activity. Intriguingly, we detected a CxCx motif near the conserved DYW tripeptide or its variants, which may be crucial for protein functions. These results indicate that the DYW domain may have multiple functions.

**Fig 2:**
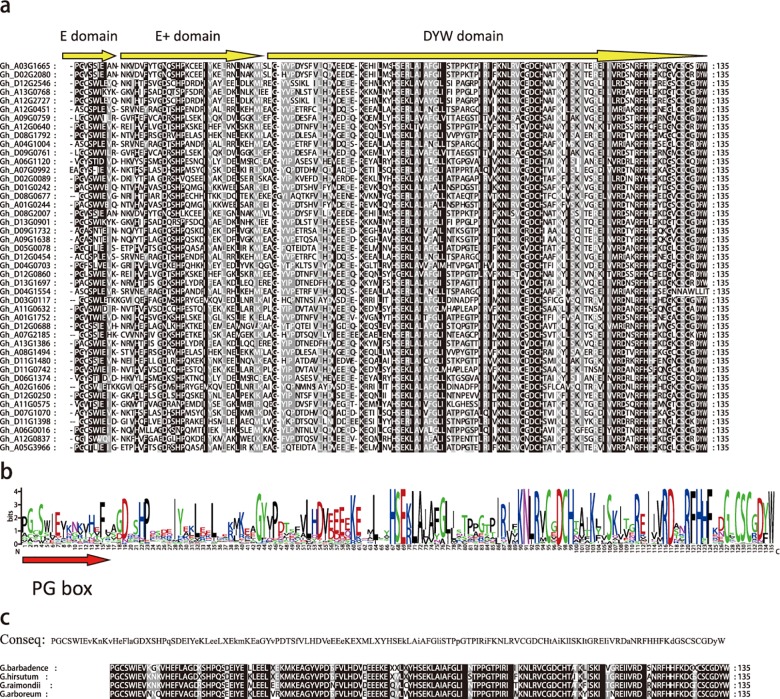
a: The alignment of the DYW deaminase domain in *G*. *hirsutum*; b: The Logo of DYW deaminase domain in *G*. *hirsutum*; c: The consensus sequence in *G*. *hirsutum* and comparison of DYW deaminase domain in *Gossypium*. The capital letters represent highly conserved, lowercase letters indicate lower conservative.

We also obtained the complete consensus sequence of the *G*. *hirsutum* DYW deaminase domain containing part of the E domain, the whole E+ domain, and the DYW domain (Fig 2). We compared this sequence with the DYW deaminase domain sequences from other three cotton species. Furthermore, we obtained a conserved PG box sequence of the E and E+ domains as described by Hayes et al. (2013). The PG box may bind to different factors to generate editing components, although the role of the PG box in editing events has not been clarified [21]. The intact DYW deaminase domain sequences were highly similar among the different analyzed cotton species, and contained the PG box, HxEx…CxxCH, and CxCxDYW motifs. These results indicate the DYW deaminase and DYW domains may exhibit similar molecular characteristics and functions.

### Chromosomal localization and evolutionary relationships among *Gossypium* DYW deaminase genes

**Fig 3:**
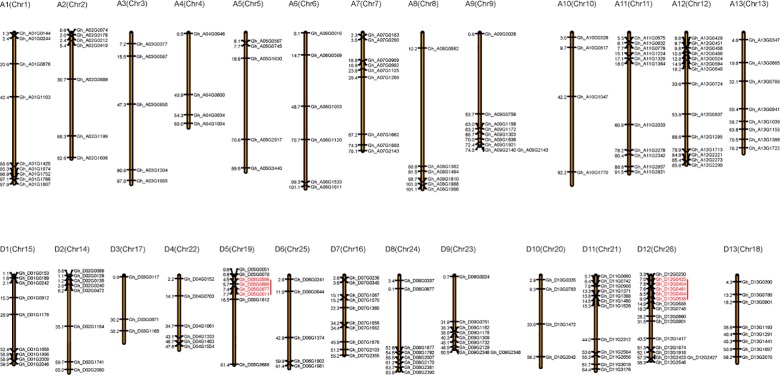
Mapping of the DYW deaminase genes in the chromosomes. Partial DYW deaminase genes localized in scaffolds.

Of the identified 227 *G*. *hirsutum* DYW deaminase genes, 203 genes were mapped to chromosomes, with 97 genes localized in the A subgenome and 106 genes localized in the D subgenome (Fig 3). Each chromosome contained an average of 7.8 DYW deaminase genes. In the A subgenome, chromosomes 4 and 10 carried the fewest DYW deaminase genes (i.e., only four each), while chromosome 12 contained the most (i.e., 14). In the D subgenome, chromosome 3 and 10 had the fewest DYW deaminase genes (i.e., only 3 and 4, respectively), and chromosome 12 carried the most DYW deaminase genes (i.e., 12). Furthermore, some genes clustered together on chromosomes. For example, four DYW deaminase genes localized within a 3.2-Mb region on chromosome 5 in the D subgenome (i.e., *Gh_D05G0556*, *Gh_D05G0696*, *Gh_D05G0877*, and *Gh_D05G0911*), while five DYW deaminase genes were mapped to a 2.9-Mb region on chromosome 12 of the same subgenome. Additionally, in *G*. *hirsutum*, the location of genes on chromosomes 9, 10, and 13 in the A subgenome exhibited a good collinearity with that on homoeologous chromosomes 9, 10, and 13 in the D subgenome. In contrast, the chromosomal localization of DYW deaminase genes in *G*. *raimondii* and *G*. *arboreum* did not exhibit a good collinearity with that in *G*. *hirsutum* because of differences in gene evolution (S2 Fig).

**Fig 4:**
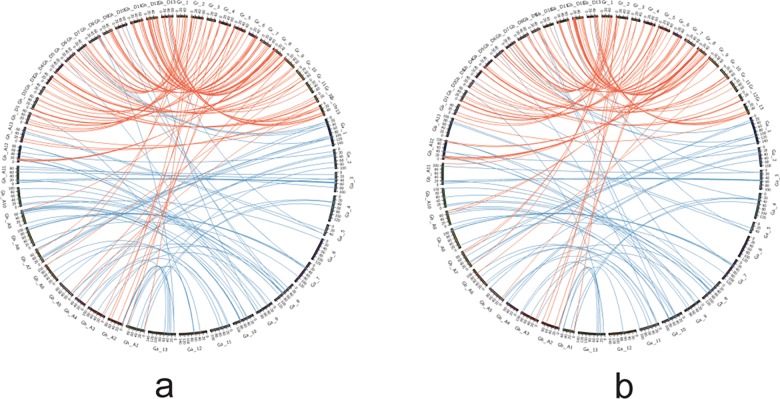
Genome wide synteny analysis of DYW deaminase genes. a: Synteny analysis between *G*. *hirsutum* and two diploid cotton species; b: Synteny analysis between *G*. *barbadense* and two diploid cotton species; Blue lines indicate syntenic regions between *G*. *arboretum* and tetraploid cotton species, red lines between *G*. *raimondii* and tetraploid cotton species.

Due to the evolutionary relationships between diploid and tetraploid cotton species, The DYW deaminases in the A and D subgenomes of the tetraploid species are homologous to those in diploid species *G*. *arboreuum* and *G*. *raimondii*, respectively. We analyzed the evolutionary relationships between the *G*. *hirsutum* and *G*. *barbadense* DYW deaminases and their corresponding proteins in *G*. *raimondii* and *G*. *arboreum*. An analysis of the DYW deaminases in the two tetraploid cotton species revealed that the *G*. *hirsutum* proteins had more tandem duplications. A comparative syntenic map of the DYW deaminase genes from tetraploid cotton species and the two diploid cotton species was constructed for a further analysis of genetic origins and evolution (Fig 4). During the evolution of *Gossypium* DYW deaminase genes, one *G*. *hirsutum* gene and seven *G*. *barbadense* genes did not have homologous genes in the two diploid cotton species (S2 Table). These genes may be new, with distinct functions in tetraploid cotton species. Additionally, 19 and 36 *G*. *raimondii* genes and 11 and 25 *G*. *arboreum* genes are not present in the *G*. *hirsutum* and *G*. *barbadense* genomes, respectively. Furthermore, 32 and 30 *G*. *raimondii* genes and 15 *G*. *arboreum* genes had more than one homologous gene in *G*. *hirsutum* and *G*. *barbadense*, respectively. We also observed that 75 and 60 *G*. *raimondii* genes and 71 and 57 *G*. *arboreum* genes each had one homologous gene in *G*. *hirsutum* and *G*. *barbadense*, respectively. Further studies are necessary to clarify the functions of the unique genes in the two tetraploid cotton species.

### Conserved DYW tripeptides and disordered amino acids in the C-terminal of DYW deaminases

In this study, we observed that the DYW and DFW tripeptides were the most frequently observed tripeptides in the C-terminal regions of DYW deaminases (Table 2). For example, there were 131 and 27 proteins that contained the DYW and DFW tripeptides in the C-terminal of *G*. *hirsutum* DYW deaminases, respectively. There were 113 and 28 *G*. *barbadense* DYW deaminases with DYW and DFW tripeptides, respectively. Of the diploid *Gossypium* species, 74, 19 and 58, 8 proteins contained the DYW and DFW tripeptide in *G*. *raimondii* and *G*. *arboretum*, respectively. Though the number of DYW tripeptides differed among *Gossypium* species, its proportions in the whole gene family were similar, ranging from 53.55% to 59.79%.

**Table 2:** Summary of conserved tripeptides in cotton and other organisms.

	*G*. *raimondii*	*G*. *arboretum*	*G*. *hirsutum*	*G*. *barbadense*	*Cyanidioschyzo Merolae*	*Physcomitrella patens*	*Selaginella moellendorffii*	*Arabidopsis thaliana*	*Oryza sativa*	*Sorghum bicolor*
Total	126	97	227	211	0	10	173	93	82	65
DYW	74	58	131	113	0	7	110	57	42	30
DFW	19	8	27	28	0	3	16	14	12	6
Disorder ^a^	17	18	37	45	0	0	15	5	17	16
DCW	1	1	2	2	0	0	10	0	0	0
CXCX_	0	0	0	0	0	0	8	1	0	1
DHW	1	2	3	2	0	0	6	1	0	1
DYC	2	1	4	5	0	0	2	0	0	0
NIW	0	0	0	0	0	0	2	0	0	0
DTW	0	0	0	0	0	0	2	0	0	0
NFW	2	0	3	2	0	0	1	1	0	0
DIW	1	1	1	0	0	0	1	0	0	0
GYW	1	1	2	1	0	0	0	2	3	2
EYW	0	0	0	0	0	0	0	1	2	2
GFW	1	1	2	2	0	0	0	2	1	1
CXCXG_ _	1	1	2	1	0	0	0	0	1	1
EFW	0	0	0	0	0	0	0	1	1	1
NYW	0	0	1	1	0	0	0	1	1	1
CXCXD_ _	0	1	0	0	0	0	0	1	0	1
DMW	0	0	0	0	0	0	0	0	1	0
DYG	2	1	2	2	0	0	0	0	0	0
DLW	1	0	1	1	0	0	0	1	0	0
DSW	1	1	2	0	0	0	0	2	0	0
DKW	1	0	2	2	0	0	0	0	0	0
DVE	1	0	2	2	0	0	0	0	0	0
DRW	0	0	0	0	0	0	0	1	0	0
NLW	0	0	0	0	0	0	0	1	0	0
DNW	0	1	0	0	0	0	0	0	0	0
GLW	0	1	1	1	0	0	0	0	0	0
YFW	0	0	1	0	0	0	0	0	0	0
DIC	0	0	1	0	0	0	0	0	0	0
DYR	0	0	0	1	0	0	0	0	0	0
DFC	0	0	0	0	0	0	0	1	0	0
NCW	0	0	0	0	0	0	0	0	0	1
DAW	0	0	0	0	0	0	0	0	0	1
DFG	0	0	0	0	0	0	0	0	1	0

^a^ Disorder means disordered amino acid residues contained in C-terminal.

The types and number of tripeptides present in the C-terminal of DYW deaminases vary depending on organisms, including *Cyanidioschyzon merolae*, *Physcomitrella patens*, *Selaginella moellendorffii*, *A*. *thaliana*, *Oryza sativa*, and *Sorghum bicolor* (Table 2). Interestingly, some sequences contained other types of C-terminal tripeptides (with disordered amino acid residues), accounting for different proportions of the tripeptides in diverse organisms. For example, there are no DYW deaminases in *C*. *merolae*, while *P*. *patens* contained only DYW deaminases with C-terminal DYW and DFW tripeptides. However, 15 DYW deaminases in *S*. *moellendorffii* (i.e., 8.67%) contained a disordered amino acid residue in the C-terminal region. Additionally, 17.7% and 5.38% of the *Gossypium* species and *A*. *thaliana* DYW deaminases contained a disordered amino acid residue, respectively.

### Subcellular localization and gene ontology analysis of DYW deaminases

To predict the subcellular locations of DYW deaminases, the Target P online program was used to examine protein transport. The DYW deaminases were detected in similar subcellular locations in all four analyzed cotton species. In *G*. *hirsutum*, 120 DYW deaminases (i.e., 53%) were localized to the mitochondria and chloroplasts, while 16 proteins (i.e., 7%) containing an N-terminal signal peptide were transported to other locations. However, we were unable to predict the subcellular locations of 40% of the predicted DYW deaminases, suggesting the need for further investigations. In other cotton species, the distribution of DYW deaminases in specific subcellular locations was similar to that of *G*. *hirsutum* (S3 Fig).

**Fig 5:**
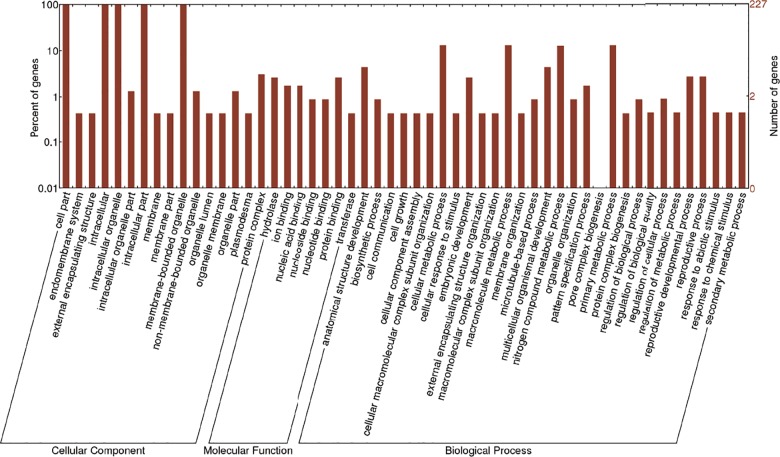
The GO analysis of DYW deaminase proteins in *G*. *hirsutum*.

Gene ontology (GO) analysis revealed that *G*. *hirsutum* DYW deaminases may be important for developmental processes (Fig 5). The cellular component proteins were mainly associated with intracellular or membrane-bound organelles, which is consistent with the subcellular localization results for DYW deaminases. The primary molecular functions included binding as well as hydrolase and transferase activities. The main biological process terms were related to cellular metabolic processes, macromolecule metabolic processes, nitrogen compound metabolic processes, and primary metabolic processes.

### Phylogenetic of *Gossypium* DYW deaminases

**Fig 6:**
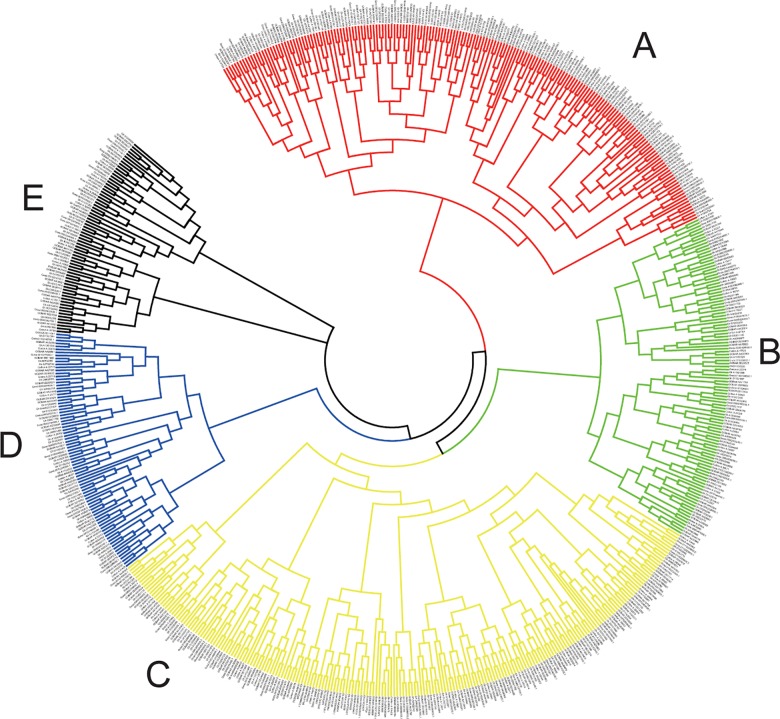
Phylogenetic tree of DYW deaminase genes in *Gossypium*.

To clarify the evolutionary relationships among *Gossypium* DYW deaminases, we constructed a phylogenetic tree using the MEGA 6 program (Fig 6). The DYW deaminases were divided into five groups (i.e., A–E) based on protein sequence similarities, with groups C and E containing the most and fewest proteins, respectively.

### *Gossypium* DYW deaminase gene expression patterns at different developmental stages and tissues

**Fig 7:**
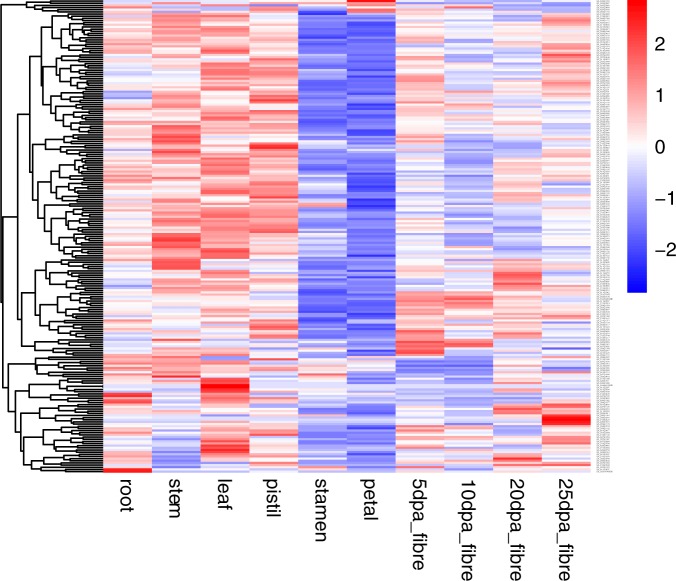
Expression profiles of DYW deaminase genes during fiber development and in different tissues. The color bar represents the expression values.

To understand the temporal and spatial expression patterns of these PPR DYW deaminase genes in Upland cotton, a publicly deposited RNA-seq data were used to assess the expression across tissues and developmental stages. Among the 227 PPR DYW deaminase genes, there were 219 genes with an FPKM>0 in at least one of the 10 investigated tissues and developmental stages (Fig 7). The expression for the remaining 8 genes was not detected and will not be discussed in this study. We observed that the expression of DYW deaminase domain-containing PPR genes were different in specific tissues and developmental stages. For example, most of the genes were up regulated in root, stem, leaf and pistil, and fiber tissues, while they were down regulated in petal and stamen tissues except for *Gh_A12G0429*, *Gh_A13G1155*, *Gh_D08G0677* and *Gh_D12G2546*. Some genes were specifically expressed in different tissues. For example, *Gh_A13G1722* gene was petal-specific, while *Gh_D05G3688* was root-specific, because they were highly expressed in petal and root, respectively.

### Quantitative real-time PCR (qRT-PCR) analysis

**Fig 8:**
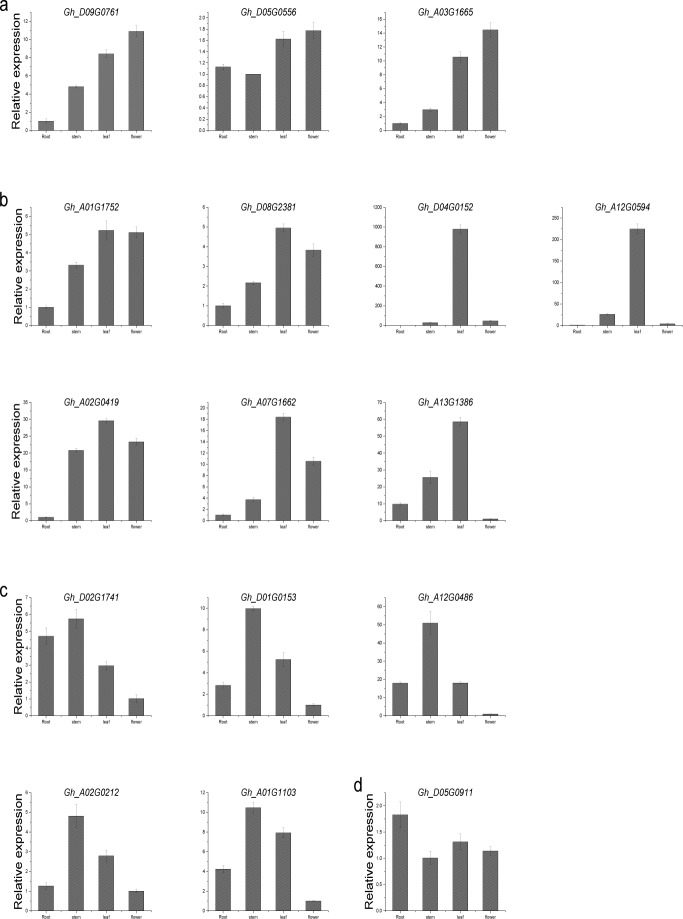
Expression analysis of 16 selected DYW deaminase genes in different tissues through qRT-PCR. a: High expression in the flower; b: Expression peaks in the leaf; c: High expression in the stem; d: High expression in the root.

A quantitative real-time PCR was used to investigate the expression levels of 16 randomly selected PPR DYW deaminase genes in roots, stem, leaves, and flowers of Upland cotton (Fig 8). All of the 16 selected genes were expressed in the four analyzed tissues, with different tissue-specific expression profiles. Among these genes, *Gh_D09G0761*, *Gh_D05G0556*, and *Gh_A03G1665* were the most highly expressed genes in flowers. *Gh_D09G0761* and *Gh_A03G1665* produced similar expression profiles (i.e., gradual increase in the four examined tissues). The results imply that these genes may have a special role during flower development. *Gh_A01G1752*, *Gh_D08G2381*, *Gh_D04G0152*, *Gh_A12G0594*, *Gh_A02G0419*, *Gh_A07G1662*, and *Gh_A13G1386* were the most highly expressed genes in leaves. Additionally, highest expression levels for *Gh_D02G1741*, *Gh_D01G0153*, *Gh_A12G0486*, *Gh_A01G1103*, and *Gh_A02G0212* were observed in stems. Finally, *Gh_D05G0911* was highly expressed in the roots. These results indicate that DYW deaminases may be important for cotton development.

### Differentially expressed PPR genes between CMS-D2 and restorer

**Fig 9:**
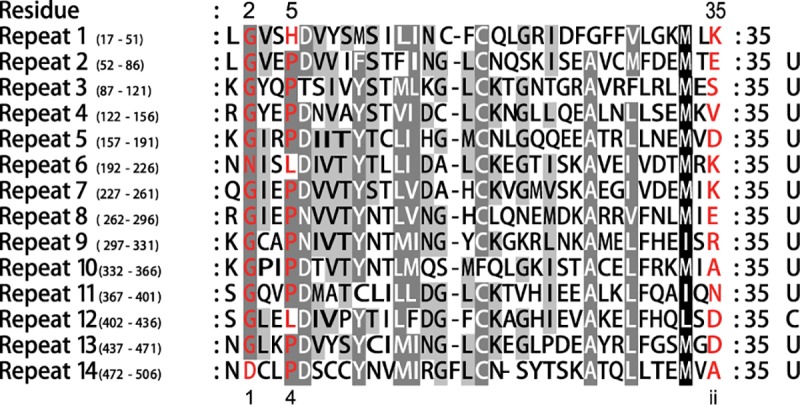
Sequence alignment of the 14 repeats of Gh_D05G3392. The residues at 2, 5 and 35 positions were predicted to be the molecular determinants for RNA binding specificity are red. The RNA sequences recognized are listed on the right, 5’ to 3’ from top to bottom.

In a genomewide transcriptome analysis of floral buds between near-isogenic CMS-D2 (i.e., A line) and its restorer (R line) differing in the *Rf1* locus, 6 differentially expressed (DE) PPR genes were identified (S1 Table). However, no DYW deaminase domain in C-terminal was found in these 6 PPR genes. Among these 6 PPR genes, *Gh_A02G1102* (PLS subgroup), *Gh_A11G0734* and *Gh_D11G0852* (E subgroup) belong to the PLS subfamily, while *Gh_A06G0542*, *Gh_D05G3392* and *Gh_D06G0610* belong to the P subfamily. It is interesting to note that, as compared with the R line, 3 PLS subfamily genes (*Gh_A02G1102*, *Gh_A11G0734* and *Gh_D11G0852*) were up regulated and 3 P subfamily genes (*Gh_A06G0542*, *Gh_D05G3392* and *Gh_D06G0610*) were down regulated in the A line. Furthermore, the PPR gene *Gh_D05G3392* is located on the chromosome where the restorer gene *Rf1* resides. The Gh_D05G3392 protein belongs to P subfamily and contain 14 repeats of PPR motifs. According to the previous studies [22–26], we adopted the three number PPR code as proposed by Yin et al [24] and predicted the amino acids in 3 particular positions (1, 4 and ii; residues 2, 5 and 35, respectively, as used in this study) within Gh_D05G3392 protein motifs. In Fig 9, we aligned the PPR motif and highlighted the residues at 2, 5 and 35 positions. Additionally, we also predicted the RNA sequence (UUUUUUUUUUCUU) recognized by the Gh_D05G3392 according to the rule proposed by Yagi et al [23]. Interestingly, one of the blast results in NCBI showed that this sequence (UUUUUUUUUUCUU) is a partial sequence of a pseudogene in *G*. *hirsutum* and SSR marker GNCOT-C1-F (GenBank: KX090570.1). However, it is outside of the *Rf1* locus [27], while other five genes were even not located on the *Rf1*-carrying chromosome (Gh_D05). Thus, with the introduction of the dominant restorer gene *Rf1* to the A line containing the CMS-inducing cytoplasm, the expression of the three PLS subfamily genes (PLS and E subgroup) was suppressed, while other three genes in the P subfamily were activated for male fertility restoration in the R line. These results indicated that DYW deaminase domain-containing PPR genes may not directly function in the CMS occurrence or fertility restoration. This is consistent with previous results in cloned restorer genes from other plant species.

## Discussion

### Relationship between DYW deaminase and DYW domains

In this study, we identified 227 DYW deaminase genes in the *G*. *hirsutum* genome. We also obtained the consensus sequence of the *Gossypium* DYW deaminase domain. Compared with the DYW domain, the complete DYW deaminase domain contained the E and E+ domains, which was consistent with the findings of a previous study [7]. Additionally, a PG-box was detected in the DYW deaminase domain, spanning the E and E+ domains. The functions of this PG box have not been characterized.

### Molecular evolution of DYW deaminases

Allotetraploid *Gossypium* species evolved from two diploid cotton species through hybridizations and genome doubling that occurred 1–2 million years ago [11]. Therefore, diploid and allotetraploid cotton species are useful models for investigating the evolution of plants. The whole genome sequences of *Gossypium* species have enabled researchers to study the molecular evolutionary characteristics of *Gossypium* DYW deaminases. In this study, we analyzed the types and number of DYW tripeptides or their variants in four *Gossypium* species (Table 2). We observed that DYW tripeptides are the most conserved and abundant, followed by the DFW tripeptide. Additionally, some tripeptide types are specific to different *Gossypium* species. Furthermore, in contrast to the diploid species, the number of tripeptide types increased and the proportion of conserved tripeptides in the whole DYW deaminase family decreased in allotetraploid *Gossypium* species.

By comparing the types and number of tripeptides present in the C-terminal of DYW deaminases from red algae (*C*. *merolae*), bryophytes (*P*. *patens*), lycophyta (*S*. *moellendorffii*), monocots (*Sorghum bicolor* and *Oryza sativa*), and eudicots (*A*. *thaliana* and *Gossypium* species), we determined that diverse plant species evolved differently in terms of the types and number of tripeptides. For example, *C*. *merolae* lacks DYW deaminases, while *P*. *patens* produces 10 DYW deaminases with a conserved C-terminal tripeptide (i.e., either DYW or DFW). *Selaginella moellendorffii* rapidly evolved 10 types of tripeptides, and the proportion of conserved DYW tripeptides decreased. Additionally, a disordered amino acid residue is present in the C-terminal of some *S*. *moellendorffii* DYW deaminases, similar to corresponding DYW deaminases in monocots and eudicots. This indicates that the types of conserved DYW tripeptide increased during the evolution from algae to angiosperms. Selection pressures may have induced the adaptation of DYW tripeptide types to evolutionary processes. Previous studies indicated that the phylogenetic distribution of DYW domains is highly correlated with RNA editing, with the number of RNA editing events increasing during evolution [6, 28]. Thus, the types and number of conserved tripeptides in DYW domains may be highly correlated with RNA editing events.

The frequency of residue changes differs among conserved tripeptides. In the DYW tripeptide, Trp is the most conserved residue, followed by Asp. We observed that most DYW deaminases contain a CxCx motif near the conserved tripeptide. However, some protein sequences lacking the CxCx motif may also be missing the HxExx…CxxCH motif. This may be because during early plant evolutionary processes, the conserved DYW tripeptide sequence became flexible and distinct tripeptides due to residue changes increased the number of tripeptide types. Some flexible tripeptides were gradually lost during evolution, which resulted in the loss of the CxCx motif and other sequences in the DYW domain following the tripeptide. Based on the relationship between the conserved DYW tripeptide and CxCx motif, we hypothesized that the conserved DYW tripeptide functions like a protective cap to prevent the loss of the CxCx motif. As distinct parts of DYW deaminases, the roles of the CxCx motif and conserved DYW tripeptide should be more thoroughly characterized in future studies.

### PPR gene family and male fertility restoration

The PPR gene family constitutes a large family of RNA binding in plants, it involves in many cellular functions and biological processes in organelles including gene expression, RNA stabilization, RNA cleavage and RNA editing [25, 29]. Previous studies indicated the PPR proteins involved in RNA editing events mostly present a characteristic motif, which contain a PPR tract followed by C-terminal motifs (E, E+ and DYW), although the E+ and DYW domain are often missing [30]. Additionally, a conserved motif HxE(x)nCxxCH exist in DYW domain is required for C to U conversion, rather than for recognition the editing site [31]. In this study, we found the complete DYW deaminase domain contained the part of E, E+ and DYW domains, and most DYW deaminases contain a CxCx motif near the conserved tripeptide. However, some protein sequences lacking the CxCx motif may also be missing the whole or part of HxExx…CxxCH motif. The DYW deaminase domain as the catalytic domain maybe promote C to U conversion. As the tandem arrays of PPR motifs, Yagi et al. (2013) found amino acid residues at 3 particular position (1, 4, and ii) within the PPR motif recognizing the upstream nucleotide sequence for the editing sites [23]. Furthermore, Takenaka et al. (2013) found that combining the P, L and S motifs will improve the prediction of RNA editing target sites [26]. In this study, we predicted the PPR code in 3 particular positions (1, 4 and ii; residues 2, 5 and 35, respectively, as used in this study) within Gh_D05G3392 motifs and predicted the RNA sequence (UUUUUUUUUUCUU) recognized by Gh_D05G3392, which are part of a pseudogene in *Gossypium hirsutum* and SSR marker GNCOT-C1-F (GenBank: KX090570.1).

CMS in flowering plants is characterized by a maternally inherited trait that lacks functional pollen grains [32], and this trait can be restored by nuclear genes known as restorer-of-fertility (*Rf*) genes [33]. Previous studies indicated that RNA editing events may have certain relationship with CMS occurrence in many crops. For example, Wei et al. (2008) found that the editing of atp9 change an arginine codon into a termination codon, shorting the protein to the standard in Ying xiang B while in Ying xiang A, which has no termination codon, cannot be a normal protein [34]. Wang et al. (2009) found that RNA editing occurrence rates in atp6 and cox2 generally increase in sterile lines [35]; Chakraborty et al. (2015) indicated that an over-expression of the unedited mitochondrial orfB gene generated male sterility in fertile rice lines in a dose-dependent manner, in which the unedited orfB gene expression increased, resulting in a low level of ATP synthase activity of F1F0-ATP synthase (complex V) [36]. In cotton, Suzuki et al. (2013) identified RNA editing sites in eight mitochondrial genes among CMS-D8 three line systems, and found that the restorer gene may change RNA editing efficiency [37]. However, little is known about the RNA editing events of mitochondrial genes in CMS-D2. Wu et al. (2011) found a C-U and a C-G editing site at position 1178 and 1214 bp downstream of the ATG codon in atpA and a A-U and a C-U editing site at position 18 and 35 bp upstream of ATG codon in nad6 between maintainer and CMS-D2 lines, respectively [16]. Thus, we speculate that RNA editing events in D2 mitochondrial genes may regulate ATP synthase activity, and the impaired ATP synthesis results in CMS occurrence.

Fertility restoration generally requires the reduction of CMS-associated RNAs or proteins by the action of the *Rf* gene (s). Most of the restorer genes cloned so far are reported to encode a PPR protein. For instance, the radish *Rfo* [38], and two *PPR* genes in sorghum [14, 15], and *Rf4* and *Rf6* gene in rice [12, 13] each encodes a different PPR protein. Additionally, previous studies indicated that most of them belong to P subfamily, except for that fact that the *Rf1* gene in sorghum encoded a PPR protein which is classified as member of E subgroup of PLS subfamily [14, 39][14, 33]. In this study, 6 differentially expressed (DE) PPR genes were identified through a genomewide transcriptome analysis between A and R lines. Interestingly, 3 PLS subfamily genes (*Gh_A02G1102*, *Gh_A11G0734* and *Gh_D11G0852*) were up regulated and 3 P subfamily genes (*Gh_A06G0542*, *Gh_D05G3392* and *Gh_D06G0610*) were down regulated in the A line. These results indicated that DYW deaminase domain-containing PPR genes may not directly function in the CMS occurrence or fertility restoration, while P subfamily genes might have critical role in the process of fertility restoration. And this is consistent with the previous studies that most of *Rf* genes belong to P subfamily [39]. Previous studies indicated a small subgroup of PPR genes existed in plant genomes that share high similarity with the identified Rf-PPRs, called as Rf-like PPRs (*RFL*) genes [38, 40]. For example, in radish *Rfo* loci, three similar PPRs were identified, which may arise through gene duplication events [38]. In this study, five genes were not located on the *Rf1*-carrying chromosome (Gh_D05), while the PPR gene *Gh_D05G3392* is located on the chromosome where the restorer gene *Rf1* resides [27], though it is outside of the *Rf1* locus and its protein was predicted to target in the chloroplasts, and the physical distance is short. Therefore, we speculate that the *Gh_D05G3392* gene may share a high similarity with *Rf1* in cotton but it is not the candidate gene for *Rf1*. As for the mechanism of CMS restoration in cotton, due to the limited study, we speculate that the *Rf1* may be a PPR gene belonging to the P subfamily. Takenaka et al. (2013) proposed that the P subfamily PPR proteins often bind tightly to their RNA targets and cannot be removed [26]. And Suzuki et al. (2013) indicated that the restorer gene may alter RNA editing efficiency among the CMS-D8 three line systems [37]. Thus, Rf1-PPR protein maybe binds tightly to their CMS gene or product, resulting in that the DYW deaminase gene cannot edit the CMS gene product, so the male fertility is restored. However, the RNA editing events in CMS-D2 cotton and the role of *Rf1* gene in RNA editing still need further studies.

## Conclusion

We identified DYW deaminases based on four sequenced *Gossypium* species genomes. We conducted chromosomal and subcellular localization experiments as well as gene structure, expression, and GO analyses for the identified genes and proteins. Most of the *Gossypium* DYW deaminases contained the conserved Asp-Tyr-Trp tripeptide (or variants) in the C terminal. The DYW deaminases lacking the conserved tripeptide may have been gradually lost during evolution. The PPR proteins containing the DYW domain are ancient RNA-editing proteins. With increasing numbers of editing sites, some amino acids in the DYW domain were gradually lost during evolution, resulting in an increase in the abundance of proteins from other subgroups (e.g., when the DYW domain was converted to an E domain). Therefore, the DYW deaminase domain might influence nucleotide deamination, which is consistent with the predicted molecular function based on GO analysis. Furthermore, regarding subcellular localizations, DYW deaminases were predicted to mainly localize in mitochondria or chloroplasts, which was consistent with GO analysis results related to cell components. In addition, the transcriptome analysis indicated no differentially expressed DYW deaminase-coding PPRs were directly associated with male sterility and restoration in the CMS-D2 system. In summary, our study provides basic information regarding the structural and evolutionary characteristics of DYW deaminases in cotton. Our findings may be useful for improving cotton production in future studies.

## Supporting information

S1 Fig The number of introns in DYW deaminase genes in *Gossypium*. (TIF)

S2 Fig The gene structure of DYW deaminase in *Gossypium*. (EPS)

S3 Fig Mapping of the DYW deaminase genes in the chromosomes of *G*. *arboretum*, *G*. *raimondii* and *G*. *barbadense*. (EPS)

S4 Fig Subcellular localization of DYW deaminases in *Gossypium*. (TIF)

S1 Table Differentially expressed genes between CMS-D2 and restorer. (XLSX)

S2 Table Information about the DYW deaminase genes in *Gossypium*. (XLSX)
