# Supplementary figures and images for: A genome-wide identification and analysis of the DYW-deaminase genes in the pentatricopeptide repeat gene family in cotton (Gossypium spp.)
Source: PLoS One. 2017 Mar 24;12(3):e0174201. doi: 10.1371/journal.pone.0174201 (PMC5365158; doi:10.1371/journal.pone.0174201)

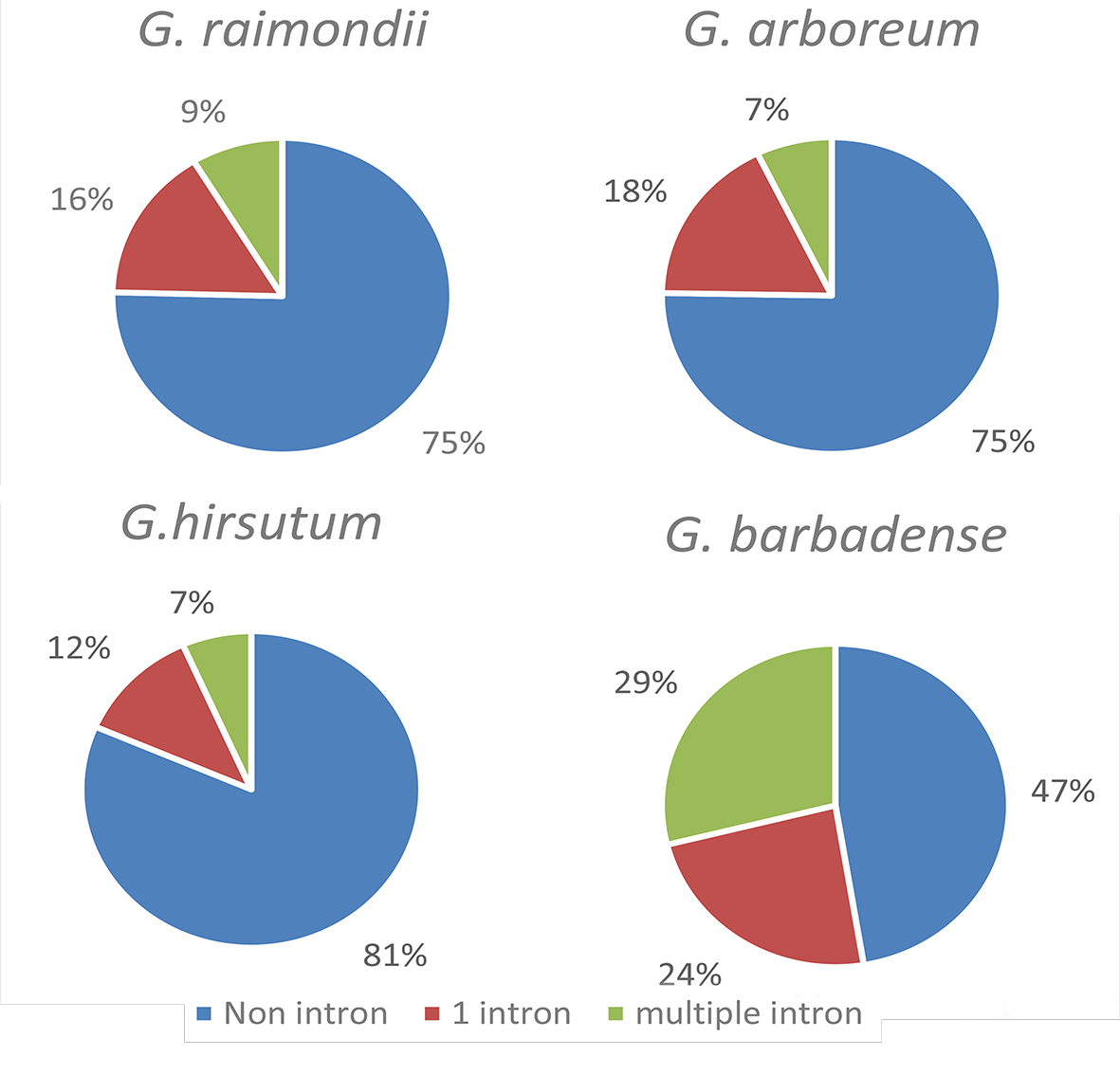

Supplement: S1 Fig — (TIF) [file pone.0174201.s001.tif]

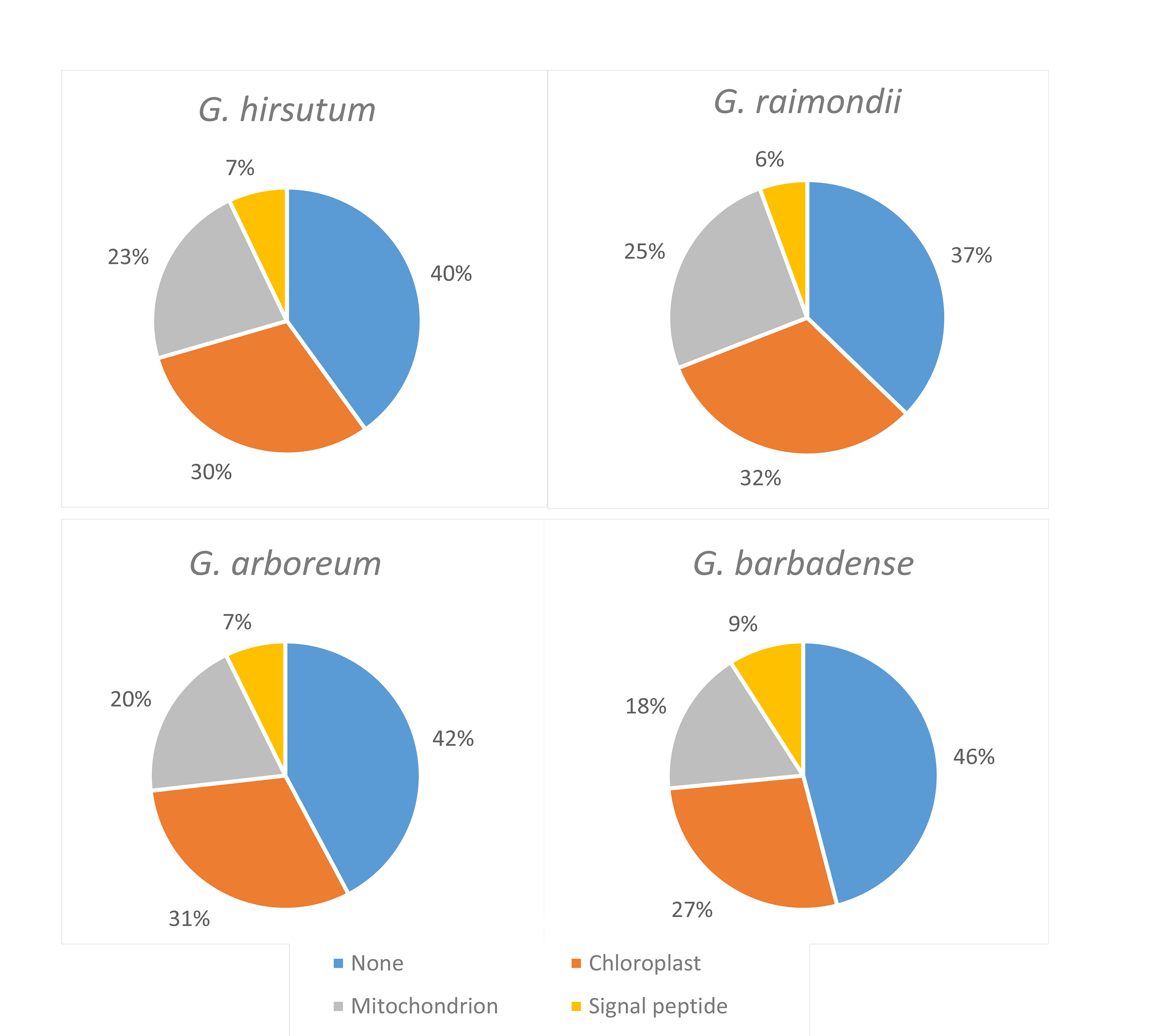

Supplement: S4 Fig — (TIF) [file pone.0174201.s004.tif]
